# The impact of frailty on admission to home care services and nursing homes: eight-year follow-up of a community-dwelling, older adult, Spanish cohort

**DOI:** 10.1186/s12877-020-01683-9

**Published:** 2020-08-06

**Authors:** Francisco Cegri, Francesc Orfila, Rosa M. Abellana, María Pastor-Valero

**Affiliations:** 1 Centre d’Atenció Primària Sant Martí, Gerència Territorial de Barcelona, Institut Català de la Salut, Barcelona, Spain; 2 Unitat de Suport a la Recerca Barcelona, Fundació Institut Universitari per a la recerca a l’Atenció Primària de Salut Jordi Gol i Gurina (IDIAPJGol), IDIAP Jordi Gol. Gran Via Corts Catalanes 587, Àtic., 08007 Barcelona, Spain; 3 Gerència Territorial de Barcelona, Institut Català de la Salut, Barcelona, Spain; 4 Department of Clinical Foundations, Faculty of Medicine, Barcelona University, Barcelona, Spain; 5 Department of Public Health History of Science and Gynecology, Miguel Hernández University of Elche, Alicante, Spain; 6 Center for Biomedical Research in Epidemiology and Public Health Network (CIBERESP), Madrid, Spain

**Keywords:** Cohort study, Frail elderly, Primary health care, Risk prediction models, Long-term home care, Long-term institutional care

## Abstract

**Background:**

Frailty in older adults is a common multidimensional clinical entity, a state of vulnerability to stressors that increases the risk of adverse outcomes such as functional decline, institutionalization or death. The aim of this study is to identify the factors that anticipate the future inclusion of community-dwelling individuals aged ≥70 years in home care programmes (HC) and nursing homes (NH), and to develop the corresponding prediction models.

**Methods:**

A prospective cohort study was conducted in 23 primary healthcare centers located in Catalonia, Spain, with an eight-year follow-up (2005–2013). The cohort was made up of 616 individuals. Data collection included a baseline multidimensional assessment carried out by primary health care professionals. Outcome variables were collected during follow-up by consulting electronic healthcare records, and the Central Registry of Catalonia for mortality. A prognostic index for a HC and NH at 8 years was estimated for each patient. Death prior to these events was considered a competing risk event, and Fine–Gray regression models were used.

**Results:**

At baseline, mean age was 76.4 years and 55.5% were women. During follow-up, 19.2% entered a HC program, 8.2% a NH, and 15.4% died without presenting an event. Of those who entered a NH, 31.5% had previously been in a HC program.

Multivariate models for a HC and NH showed that the risk of a HC entry was associated with older age, dependence on the Instrumental Activities of Daily Living, and slow gait measured by Timed-up-and-go test. An increased risk of being admitted to a NH was associated with older age, dependence on the Instrumental Activities of Daily Living, number of prescriptions, and the presence of social risk.

**Conclusions:**

Prognostic models based on comprehensive geriatric assessments can predict the need for the commencement of HC and NH admission in community-dwelling older adults. Our findings underline the necessity to measure functional capacity, mobility, number of prescriptions, and social aspects of older adults in primary healthcare centers. In such a setting they can be offered longitudinal holistic assessments so as to benefit from preventive actions in order to remain independent in the community for as long as possible.

## Background

Worldwide, progressive population aging presents increasingly multiple health, social, and economic consequences for systems with inadequate planning and resources. This demographic change is leading to an augmented prevalence of chronic diseases and frailty in older adults, resulting in loss of autonomy and placing a heavier burden on health and social care [1–4].

In Spain, 17% of the inhabitants are currently > 65 years, and projections for 2050 indicate that the figure will reach 33% compared to the expected 29% in neighbouring European countries [4]. Many older individuals are, or will become, frail. Such a condition in the elderly is a multidimensional clinical entity that represents a state of vulnerability to stressors [5], including a reduction in physical, mental, and social functions, and predicts adverse events such as hospitalization [6], institutionalization [7], and death [8].

There are different ways to measure frailty, the most common measurements are the frailty phenotype [9] and the deficit accumulation model [10]. Other tools include performance tests (such as the Short Physical Performance Battery and the Timed-Up-and-go Test) and scales that assess the instrumental activities of daily life [11, 12]. In addition, there is the comprehensive geriatric assessment (CGA), considered the gold standard approach for community evaluation. It is usually conducted following existing national strategies in supporting individuals living with frailty [13–15]. The CGA is a multidimensional process that can be performed in a number of healthcare settings to identify medical, social, and functional needs and develop a care plan [16, 17].

Primary Health Care (PHC), with its community perspective and longitudinal approach, is the ideal scenario for the early detection of frailty in older adults. Nevertheless, simple evaluation tests need to be incorporated into usual care practice. Early identification would allow targeted support from health and social services to help older individuals improve their quality of life and live autonomously in their homes for as long as possible. In addition to respecting the preferences of most of them [18], it would provide a more cost-effective alternative to institutionalization.

Adverse outcomes in the progression of frailty include dependency and institutionalization. In Spain, one of the primary care resources available for these individuals is the Home Care programme (HC). It offers comprehensive, continuous health and social attention to individuals in situations of functional decline and/or dependency who cannot attend health centers with the aim of keeping them as long as possible in their home with the corresponding autonomy. The HC programmes offered by the Spanish PHC cover some 6% of individuals > 65 years, the population generally considered to be at a state of advanced frailty or disability [19].

Nevertheless, as frailty advances the complexity of medical attention augments and the caregiver and/or social support may have difficulties maintaining the quality of care. At this point, nursing home (NH) services become the most appropriate resource, despite the considerable financial outlay for both the public health system and the family economy. In our region NH services cover 3% of those > 65 years of age [20].

Most studies have focused on validating instruments to identify and describe the characteristics of frail older individuals [11] whilst other authors have established the determinants of HC and NH use [21–23]. The aim of this study is to characterize the factors that will lead to the future inclusion of older, community-dwelling subjects into HC programmes/NH institutionalization, and to develop the corresponding risk prediction models. Both events are related to the progression of frailty, functional decline, and loss of autonomy all of which could be delayed/prevented by an adequate and timely approach to those at risk. These individuals can then benefit from personalized preventive actions in order to remain independent in the community for as long as possible.

## Methods

### Study design and setting

We conducted an eight-year follow-up, multicentre, cohort study in 23 PHC centers in Catalonia, Spain. The 23 PHC were not randomly selected, although special care was taken to include PHC with different socioeconomic status as well as urban and rural health centers. Out of the 50 PHC which were invited to participate, a final sample of 23 accepted (20 in urban areas and 3 in rural ones). These PHC belong to the National Health System in Catalonia and are distributed geographically in 3 of the 4 Catalonia provinces, (Barcelona, Lleida and Girona). They serve an assigned population, which varies from 1179 to 29,214 individuals from middle-low to middle-high socioeconomic status. The inclusion period was from October 2004 to June 2005.

### Participants

The cohort was made up of a random sample of 616 subjects ≥70 years of age. Interview participation was turned down by 75 individuals (73% women) with no age differences compared to the study members. Exclusion criteria included subjects already receiving HC and NH services, those presenting severe mental disorders/terminal illness, and non-residents in the reference area.

### Data collection and study variables

Subjects were randomly selected from the daily schedule of the healthcare professionals taking part in the study, and were included once the informed consent was signed.

Participants were programmed for the interview and the CGA in their corresponding PHC center. The healthcare professionals (nurses and doctors) carried out both the interview and the CGA. The main information collected in interviews through standardized questionnaires included sociodemographic data, physical activity, body mass index (BMI), self-reported health [24], number of hospitalization and falls in the previous year, and prescribed drugs.

The CGA included:

*Functional Assessment:*

- Basic Activities of Daily Living (BADL) with the Barthel Index [25] (from 0 to 100 points), < 60 represents moderate/severe dependence.
- Instrumental Activities of Daily Living (IADL) with the Lawton and Brody Index [26], the cut-off points for moderate/severe dependence are < 6 points for women and < 4 points for men, respectively.
- Mobility was evaluated with the Timed-up-and-go test (TUGT) [27]. It measures in seconds the time to rise from a chair, walk a distance of 3 m, return to the chair and sit down. It includes aspects of gait, strength, balance, and speed. A score of > 10 s is usually considered as altered.

*Mental health Assessment:*

- Cognitive assessment was measured with the Lobo Mini Cognitive Examination (MCE) [28], the Spanish validated version of the Folstein Mini-mental state examination [29] (From 0 to 30 points), the cut-off point for cognitive deterioration is ≤23.
- For evaluation of the affective state, the Yesavage Geriatric Depression Scale (GDS) [30] (from 0 to 15 points) was employed with a cut-off point for probable depression of > 5.

*Biomedical assessment:*

- Nutritional status was measured using the Mini Nutritional Assessment Short Form (MNA-SF) [31] questionnaire (from 0 to 14 points), with a cut-off point ≤11 for risk of malnutrition.
- Near vision was evaluated with the Jaeger Card [32] with a cut-off point of > 20/40 for visual acuity deficit.
- Hearing was assessed with the Handicap Hearing Impairment in the Elderly Screening Version (HHIE-S) [33] (from 0 to 40 points). A cut-off point ≥10 was considered auditory limitation.
- Urinary incontinence was measured with the International Consultation on Incontinence Questionnaire Short Form (ICIQ-SF) [34] (from 0 to 21 points) with a cut-off point ≥1 for the diagnosis of urinary incontinence.
- Morbidities, selected following literature review and consensus by the original study group. The selection criteria were for those conditions most related to the development of frailty, functional limitations, and disability [35, 36]: osteoarticular diseases, cerebrovascular accident with sequelae, Parkinson’s disease, acute myocardial infarction or heart failure, chronic obstructive pulmonary disease, severe visual deficit, severe deafness, dementia, recurrent falls or fractures and chronic depression.

*Social Assessment:*

- Social vulnerability was evaluated with the Socio-Family Rating Scale of the Elderly (SFRSE) [37] (from 0 to 25 points). It assesses the family, economic situation, housing, social relations, and social support, with a cut-off point ≥10 for social risk.

We gathered the outcome variables during the eight-year follow-up by consulting the electronic primary healthcare records; telephone contacts were made in the case of incomplete information. In addition, we consulted the Central Registry of Catalonia for mortality.

Main outcome variables were:

- Inclusion in an HC program of the PHC: encoded in the electronic primary healthcare records when the service is requested by either the patient or healthcare professional, mainly due to considerable mobility difficulties.
- Inclusion in a NH: this encompasses all types of institutions such as nursing homes, long-term care institutions, private or public. It is encoded in the electronic primary healthcare records when a patient is institutionalized with the care relationship usually transferred from the PHC to the institution.
- Mortality: date of death registered in the PHC record and the Central Registry of Catalonia.

### Statistical analysis

Continuous variables were expressed as mean and standard deviation, or as median and interquartile (IQ) range, whenever appropriate. For HC and NH outcomes, death prior to these events was considered as a competing risk event, therefore the cumulative incidence function (CIF) of HC/NH risk was calculated taking it into consideration. To analyse the effect of baseline predictors for the CIF, we used the Fine–Gray [38] regression model for the sub-distribution hazard (sHR). Clinically meaningful variables showing a significant level in the univariate analysis (*P* < 0.05) were thereafter included in the multivariate model. A backward stepwise method was used to identify independent risk predictors with P < 0.05 for the inclusion or deletion criterion. The proportionality assumption of the models was verified using time-dependent variables. The discriminative ability of the models was assessed by Harrell’s C-index [39]. The internal validity of the final predictive models was tested for 150 bootstrap re-samples. The calibration of models was checked by plotting the observed and predicted probabilities of the model in groups defined by the quartiles of the predicted event probabilities. We estimated a prognostic index for home confinement and institutionalization at 8 years for each patient as the sum of the variables included in the final model multiplied by the log of the respective sHR. We classified patients using the CIF approach into three groups according to their risk of a HC/NH: low, medium, and high, by splitting the index according to tertiles. Data analysis was performed using the statistical R-3.5.1 package. The discrimination and calibration analyses were carried out with the pec package [40].

## Results

Baseline characteristics of the cohort and assessment of frailty dimensions are shown in Tables 1 and 2. The mean baseline age was 76.4 years, 55.5% were women, 73.5% did not have complete primary education, and 22% lived alone (32% women vs 9.6% men). Mean BMI was 28.5 and 4.5% smoked. Health was rated as good by 47.4% and bad by 6.7%. Patients on average took 5 drugs a day, and 32% consumed psychotropic ones. At the functional level, the mean Barthel index score was 96.5, the majority were independent for the BADL (62.5%), and only 15.3% had moderate/severe dependence. The IADL, measured with the Lawton index, showed that 76.7% were independent, and only 6.5% presented moderate/severe dependence. Mobility, measured with TUGT, was 13 s on average, and 14.4% of the participants led a sedentary life. At the mental level, the average MCE score was 27/30 points, and the GDS scores showed 19.8% probable depression.

**Table 1:** Frequency and bivariate sub-hazard ratios of admission into a Home Care programme or a Nursing Home during the 8-year follow-up in relation to the baseline characteristics of the cohort

VARIABLES	N	ALL ***N*** = 616	Death^**2**^ n = 95	HOME CARE			NURSING HOME		
				No HC ***n*** = 403	HC ***n*** = 118	Sub-hazard Ratio^**3**^ [IC95%]	No NH ***n*** = 470	NH n = 51	Sub-hazard Ratio^**3**^ [IC95%]
**Age, years, mean (SD)**	616	76.4 (4.8)	78.0 (4.9)	75.2 (4.3)	**78.9 (4.8)**	1.4 [1.1;1.2]	75.8 (4.6)	**78.7 (5.1)**	1.1 [1.1;1.2]
**Gender, female, n (%)**	616	342 (55.5%)	41 (43.2%)	233 (57.8%)	**68 (57.6%)**	1.0 [0.7;1.5]	267 (56.8%)	**34 (66.7%)**	1.5 [0.8;2.7]
**Educational level, n (%):**	599								
**Illiterate**		52 (8.6%)	7 (7.5%)	33 (8.3%)	**12 (10.3%)**	Ref.	39 (8.4%)	**6 (12.0%)**	Ref.
**Incomplete primary**		395 (65.0%)	71 (76.3%)	251 (63.1%)	**73 (62.4%)**	0.8 [0.4;1.5]	290 (62.4%)	**34 (68.0%)**	0.7 [0.3;1.8]
**Primary**		112 (18.4%)	9 (9.7%)	78 (19.6%)	**25 (21.4%)**	1.0 [0.5;2.0]	99 (21.3%)	**4 (8.0%)**	0.3 [0.1;1.1]
**Secondary**		29 (4.8%)	3 (3.2%)	24 (6.0%)	**2 (1.7%)**	0.3 [0.1;1.3]	23 (5.0%)	**3 (6.0%)**	0.8 [0.2;3.3]
**University**		11 (1.8%)	1 (1.1%)	7 (1.8%)	**3 (2.6%)**	1.2 [0.4;3.7]	9 (1.9%)	**1 (2.0%)**	0.7 [0.1;6.0]
**Living arrangements, n (%):**	613								
**Family**		167 (27.2%)	30 (31.9%)	106 (26.4%)	**31 (26.5%)**	Ref.	126 (26.9%)	**11 (21.6%)**	Ref.
**Partner**		311 (50.7%)	44 (46.8%)	206 (51.2%)	**61 (52.1%)**	1.1 [0.7;1.6]	251 (53.6%)	**16 (31.4%)**	0.8 [0.4;1.7]
**Living alone**		135 (22.0%)	20 (21.3%)	90 (22.4%)	**25 (21.4%)**	1.0 [0.6;1.8]	91 (19.4%)	**24 (47.1%)**	2.8 [1.4;5.8]
**Physical activity, n (%):**	610								
**Active (≥30 min/day)**		352 (57.7%)	37 (39.4%)	262 (65.8%)	**53 (44.9%)**	Ref.	292 (62.8%)	**23 (45.1%)**	Ref.
**< 30 min/day**		171 (28.0%)	42 (44.7%)	91 (22.9%)	**38 (32.2%)**	1.5 [1.0;2.2]	110 (23.7%)	**19 (37.3%)**	1.7 [0.9;3.1]
**Sedentary**		87 (14.3%)	15 (16.0%)	45 (11.3%)	**27 (22.9%)**	2.4 [1.5;3.8]	63 (13.5%)	**9 (17.6%)**	1.8 [0.9;3.9]
**Body mass index, mean (SD)**	592	28.5 (4.2)	28.3 (4.7)	28.2 (3.9)	**29.6 (4.4)**	1.1 [1.0;1.1]	28.5 (3.92)	**28.9 (5.1)**	1.0 [0.9;1.1]
**Body mass index, n (%):**	592								
**< 22 Low weight**		36 (6.1%)	8 (8.8%)	22 (5.7%)	**6 (5.2%)**	Ref.	22 (4.9%)	**6 (12.0%)**	Ref.
**22–26.9 Norm weight**		144 (24.3%)	26 (28.6%)	98 (25.5%)	**20 (17.2%)**	0.8 [0.3;2.0]	111 (24.6%)	**7 (14.0%)**	0.3 [0.1;0.8]
**27–29.9 Over weight**		169 (28.5%)	26 (28.6%)	96 (24.9%)	**47 (40.5%)**	1.5 [0.6;3.6]	123 (27.3%)	**20 (40.0%)**	0.7 [0.3;1.6]
**≥ 30 Obesity**		243 (41.0%)	31 (34.1%)	169 (43.9%)	**43 (37.1%)**	0.9 [0.4;2.2]	195 (43.2%)	**17 (34.0%)**	0.4 [0.1;0.9]
**Perceived health**^**1**^**, n (%):**	616								
**Good or +**		259 (42.0%)	31 (32.6%)	191 (47.4%)	**37 (31.4%)**	Ref.	211 (44.9%)	**17 (33.0%)**	Ref.
**Fair**		305 (49.5%)	54 (56.8%)	185 (45.9%)	**66 (55.9%)**	1.6 [1.1;2.4]	226 (48.1%)	**25 (49.0%)**	1.3 [0.7;2.5]
**Bad**		52 (8.4%)	10 (10.5%)	27 (6.7%)	**15 (12.7%)**	2.3 [1.3;4.2]	33 (37.0%)	**9 (17.6%)**	3.0 [1.3;6.7]
**Hospitalization/previous year, n (%), ≥1**	616	78 (12.7%)	19 (20.0%)	46 (11.4%)	**13 (11.0%)**	0.8 [0.5;1.5]	53 (11.3%)	**6 (11.8%)**	0.9 [0.4;2.1]
**Falls / previous year, n (%) ≥ 1**	615	149 (24.2%)	25 (26.3%)	101 (25.1%)	**23 (19.5%)**	0.8 [0.5;1.2]	108 (23.0%)	**16 (31.4%)**	1.5 [0.8;2.6]
**Number of drugs, mean (SD)**	582	5.0 (3.1)	6.1 (3.3)	4.6 (2.9)	**5.4 (3.1)**	1.1 [1.0;1.1]	4.6 (2.9)	**6.6 (3.2)**	1.2 [1.1;1.2]
**Psychoactive drugs, n (%), ≥1**	563	180 (32.0%)	27 (32.5%)	115 (30.7%)	**38 (36.2%)**	1.2 [0.8;1.8]	130 (30.0%)	**23 (50.0%)**	2.1 [1.2;3.8]

SD: Standard deviation; HC: Home Care; NH: Nursing Home; IC95%: 95% confidence interval

^1^Perceived health, question: “In general, would you say that your health is excellent, very good, good, fair, or bad?. ^2^Death, prior to both HC/ NH outcomes. ^3^Fine–Gray regression model for the sub-distribution hazard

**Table 2:** Frequency and bivariate sub-hazard ratios of admission into a Home Care programme or a Nursing Home during the 8-year follow-up in relation to the baseline Comprehensive Geriatric Assessment

VARIABLES	N	ALL N = 616	Death^**12**^ n = 95	HOME CARE			NURSING HOME		
				No HC n = 403	HC n = 118	Sub-hazard Ratio^**13**^ [CI95%]	No NH n = 470	NH n = 51	Sub-hazard Ratio^**13**^ [CI95%]
**BADL (Barthel**^**1**^**), mean (SD)**	616	96.5 (7.0)	95.5 (9.8)	97.5 (5.7)	**94.1 (7.7)**	1.0 [1.0;1.0]	97.1 (5.3)	**93.1 (12.1)**	1.0 [1.0;0.98]
**IADL (Lawton & Brody**^**2**^**), n (%)**	615								
**Independent**		472 (76.7%)	63 (66.3%)	336 (83.4%)	**73 (62.4%)**	Ref.	373 (79.5%)	**36 (70.6%)**	Ref.
**Mild Dependence**		103 (16.7%)	23 (24.2%)	54 (13.4%)	**26 (22.2%)**	1.7 [1.1;2.6]	73 (15.6%)	**7 (13.7%)**	0.9 [0.4;2.1]
**Moderate + Severe Dependence**		40 (6.5%)	9 (9.5%)	13 (3.2%)	**18 (15.4%)**	4.8 [2.8;8.3]	23 (4.9%)	**8 (15.7%)**	4.5 [2.0;9.9]
**Mobility assessment (Timed-up-and-go test**^**3**^**), mean (SD)**	599	13.0 (6.8)	14.6 (7.4)	11.8 (5.5)	**16.2 (9.0)**	1.1 [1.0;1.1]	12.4 (6.4)	**15.5 (8.4)**	1.1 [1.0;1.1]
**Cognitive status (MEC**^**4**^**), mean (SD)**	614	27.0 (3.7)	26.6 (3.6)	27.4 (3.6)	**26.2 (3.9)**	0.9 [0.9;1.0]	27.3 (3.6)	**25.6 (3.8)**	0.9 [0.9;1.0]
**Affective status (GDS**^**5**^**) mean (SD)**	607	3.8 (3.3)	4.2 (3.2)	3.6 (3.3)	**4.3 (3.1)**	1.1 [1.0;1.1]	3.6 (3.2)	**5.1 (3.8)**	1.1 [1.0;1.2]
**Nutritional assessment (MNA-SF**^**6**^**), mean (SD)**	604	12.9 (1.6)	12.6 (1.8)	13.0 (1.6)	**12.8 (1.6)**	0.9 [0.9;1.0]	13.0 (1.6)	**12.6 (1.8)**	0.9 [0.8;1.0]
**Visual impairment (Jaeger Card**^**7**^**), n (%)**	611	195 (31.9%)	34 (35.8%)	112 (28.0%)	**49 (42.2%)**	1.7 [1.2;2.4]	143 (30.6%)	**18 (36.7%)**	1.3 [0.7;2.4]
**Hearing impairment (HHIE-S**^**8**^**), n (%)**	612	121 (19.8%)	21 (22.1%)	71 (17.7%)	**29 (25.0%)**	1.4 [0.9;2.1]	89 (19.1%)	**11 (21.6%)**	1.2 [0.6;2.3]
**Urinary incontinence (ICIQ-SF**^**9**^**), n (%)**	615	277 (45.0%)	44 (46.3%)	163 (40.5%)	**70 (59.3%)**	1.8 [1.3; 2.7]	199 (42.4%)	**34 (66.7%)**	2.5 [1.4;4.5]
**Number of morbidities**^**10**^**, mean (SD)**	616	0.8 (0.9)	0.9 (1.1)	0.7 (0.9)	**0.9 (0.8)**	1.1 [1.0;1.3]	0.7 (0.8)	**1.0 (1.3)**	1.3 [1.1;1.6]
**Social risk (Social-familial evaluation scale**^**11**^**), mean (SD)**	614	8.8 (2.7)	8.7 (2.8)	8.7 (2.7)	**9.4 (2.7)**	1.1 [1.0;1.2]	8.7 (2.6)	**10.7 (3.2)**	1.2 [1.1;1.3]

SD: Standard deviation; HC: Home Care; NH: Nursing Home; CI95%: 95% confidence interval

^1^Basic Activities of the Daily Living (BADL) Barthel Index (from 0 to 100 points), below 60 represents moderate/ severe dependence. ^2^Instrumental Activities of the Daily Living (IADL) Lawton and Brody Index, with dependence cut-off points for women < 8 points (from 0 to 8 points) and men < 5 points (from 0 to 5 points). ^3^Timed-up-and-go test (TUGT) The score of> 10 s was considered altered. ^4^Mini Cognitive Examination (MEC), (from 0 to 30 points), cut-off point for cognitive deterioration ≤23. ^5^Geriatric Depression Scale (GDS) Yesavage Scale (from 0 to 15 points), cut-off point for probable depression> 5. ^6^Mini Nutritional Assessment Short Form (MNA-SF) (from 0 to 14 points), cut-off point ≤11 for risk of malnutrition. ^7^Jaeger Card, point > 20/40 visual acuity deficit. ^8^Handicap Hearing Impairment in the Elderly Screening Version (HHIE-S) (from 0 to 40 points) (ref). The cut-off point ≥10 was considered an auditory limitation. ^9^International Consultation on Incontinence Questionnaire Short Form (ICIQ-SF) (from 0 to 21 points) with a cut-off point ≥1 for the diagnosis of urinary incontinence. ^10^Morbidities related to frailty, including: cerebrovascular accident with sequelae, Parkinson’s disease, osteoarticular diseases, severe visual deficit, dementia, acute myocardial infarction or heart failure, chronic obstructive pulmonary disease, recurrent falls or fractures, severe deafness and chronic depression. ^11^Socio-Family Rating Scale of the Elderly (SFRSE) (from 0 to 25 points) which assesses family, economic situation, housing, social relations, and social support, with a cut-off point ≥10 for social risk. ^12^Death, previous to both HC/NH outcomes. ^13^Fine–Gray regression model for the sub-distribution hazard

**Fig. 1:**
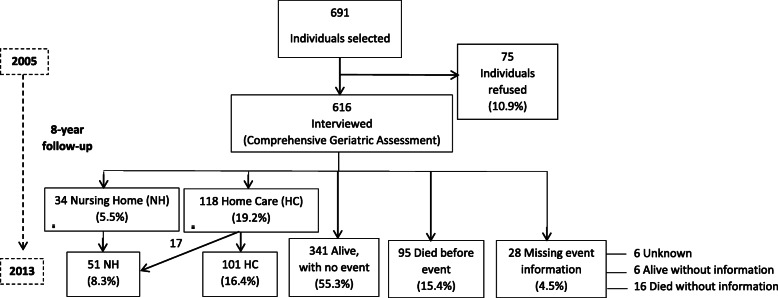
Flow diagram showing the study follow-up

During follow-up 19.2% (*n* = 118) of the 616 participants entered a HC programme (30.6 incidence per 1000 person-years), while 8.2% (*n* = 51) were admitted to a NH one (13.1 incidence per 1000 person-years). Of those admitted to a NH, 31.5% (*n* = 17) had previously been in a HC. Of the 616, mortality during follow-up was 15.4% (*n* = 95) for participants presenting no event, and 46.2% (*n* = 78) for those who were in either a HC or NH programme. During follow-up 4.5% (*n* = 28) was lost with a greater proportion of men (64%, *p* < 0.05). However, there were no statistically significant differences in the rest of the main variables between those who completed the study and those who did not (Fig. 1**).**

The median follow-up was 91.8 months (IQ: 58.1–97.7) and 92.3 months (IQ: 59.7–97.9) for the HC and NH subjects, respectively.

Table 1 shows the bivariate sHRs of admission to HC and NC programmes during the eight-year follow-up period according to baseline variables.

Comparing those who entered a HC programme with those who did not, HC incidence increased with age (78.9 years versus 75.2 years, sHR = 1.1), sedentary life style (22.9% versus 11.3%, sHR = 2.4), and poor self-perceived health (12.7% versus 6.7%, sHR = 2.3).

Table 2 shows the bivariate sHRs of admission to HC and NC programmes during the eight-year follow-up period according to the geriatric assessment.

Comparing those who entered a HC programme with those who did not, HC incidence increased with worse functional status, Barthel index (94.1 versus 97.5, sHR = 1.0), and Lawton and Brody index, in light (22.2% versus 13.4%, sHR = 1.7) and moderate dependence (15.4% versus 3.2%, sHR = 4.8). It also augmented in individuals with worse mobility (mean TUGT 16.2 versus 11.8, sHR = 1.1); worse cognitive scores (mean score 26.2 versus 27.4, sHR = 0.9); worse affective state (mean 4.3 versus 3.6, sHR = 1.1); urinary incontinence (59.3% versus 40.5%, sHR = 1.8); and higher social risk (mean 9.4 versus 8.7, sHR = 1.1).

Admission to a NH programme was associated with age (mean 78.7 versus 75.8, sHR = 1.1); living alone (47.1% versus 19.4%, sHR = 2.8); and greater drug consumption (mean 6.6 versus 4.6, sHR = 1.2).

Functional impairment in BADL was related to NH and the Barthel index (mean 93.1 versus 97.1, sHR = 1.0). Unlike a HC, however, NH entry was only associated with the highest degree of dependence in the Lawton and Brody index (moderate/severe dependence, with 15.7% versus 4.9%, sHR = 4.5). It was also related to worse mobility (longer TUGT time, mean 15.5 versus 12.4, sHR = 1.1); worse cognitive scores (mean 25.6 versus 27.3, sHR = 0.9) and risk of depression (mean 5.1 versus 3.6, sHR = 1.1).

We found a higher risk of NH admission for urinary incontinence (66.74% versus 42.4%, sHR = 2.5); higher number of specific morbidities (mean 1.0 versus 0.7, sHR = 1.3); and social risk (higher score on the socio-familial assessment scale of the elderly, mean 10.7 versus 8.7, sHR = 1.2).

A complementary, bivariate sub-analysis was carried out between the participant’s social risk and the occurrence of any events (NH, HC; data not shown). We observed a dose-response association between increasing social risk and being first admitted to a HC programme; with a still higher baseline social risk, entering a NH one: Finally, the highest baseline social risk was associated with first a HC programme and later a NH facility.

The multivariate adjusted model showed that the incidence risk of a HC entry was associated with older age, dependence on the IADL (moderate/severe dependence), and slow gait measured by TUGT. There was a significant association between the risk of being admitted to a NH programme and older age, dependence on the IADL (moderate/severe), more prescriptions, and the presence of social risk, see Table 3**.**

**Table 3:** Multivariate Competitive Risk Models for a Home Care and Nursing Home admission, prognostic index functions, and risk classification

HOME CARE (HC)			NURSING HOME (NH)		
	Sub Hazard Ratio [CI95%]	***p***-value		Sub Hazard Ratio [CI95%]	p-value
*Age (years)*	1.1 [1.1–1.2]	< 0.001	*Age (years)*	1.1 [1.0–1.2]	0.002
IADL: *Independent*	1		*IADL: Independent*	1	
IADL: *Mild Dependence*	1.5 [1.0–2.4]	0.083	*IADL: MIld Dependence*	0.6 [0.2–1.5]	0.250
IADL: *Moderate Dependence or +*	2.8 [1.4–5.6]	0.005	*IADL: Moderate Dependence or +*	2.6 [1.0–6.8]	0.045
TUGT *(seconds)*	1.0 [1.0–1.1]	0.024	*–*	–	–
*–*	–	–	*Number of drugs*	1.1 [1.0–1.2]	0.019
			*SFRSE (points)*	1.2 [1.1–1.2]	< 0.001
**PROGNOSTIC INDEX FUNCTION (PI)**					
**HC PI**			**NH PI**		
PI: 0,107*Age (years) + 0.412* Mild instrumental dependence (IADL) + 1013* Moderate instrumental dependence (includes severe and total) (IADL) + 0,0331*TUGT (seconds)			PI: 0,106*Age (years)-0.551* Mild instrumental dependence (IADL) + 0.971* Moderate instrumental dependence (includes severe and total) (IADL) + 0.097* Number of drugs + 0.165 *SFRSE (points)		
**Risk group**			**Risk group**		
•Lower risk group: PI 7.5–8.3			•Lower risk group: PI 7.9–9.5		
•Medium risk group: PI 8.4–8.9			•Medium risk group: PI 9.6–10.2		
•Higher risk: PI ≥9.0			•Higher risk: PI ≥10.3		

IADL: Instrumental Activities of Daily Living; TUGT: Timed Get Up and Go Test; SFRSE: Socio-Family Rating Scale of the Elderly; HC: Home Care; NH: Nursing Home; CI95%: 95% confidence interval

Based on the results of the multivariate analyses, we constructed two prediction models according to the risk of inclusion in a HC/NH. Subjects with a HC risk were classified into three groups: lower risk with a prognostic index (PI) between 7.5 and 8.3; medium risk, between 8.4 and 8.9; and higher risk > 9.0. The NH model was also categorized into three groups: lower risk between 7.9 and 9.5; medium risk between 9.6 and 10.2; and higher risk PI > 10.3, see Table 3.

**Fig. 2:**
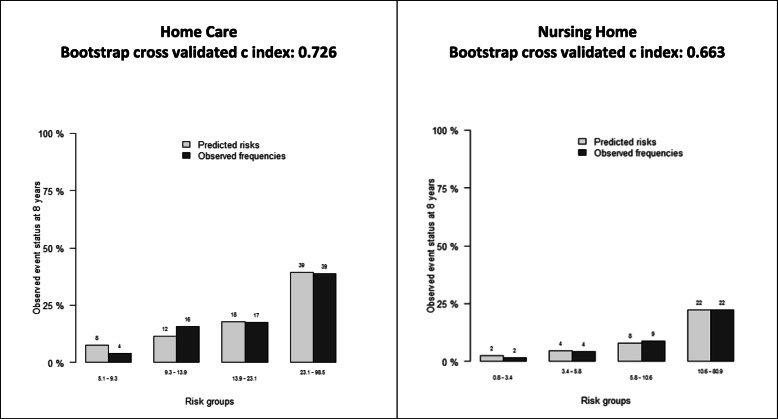
Calibration plots for risk outcome prediction and discrimination index (8 years)

The calibration plot showed that both models (HC and NH) presented a good calibration for predicting risk outcomes. In addition, discrimination was good for a HC (C-index = 0.7) and moderate for a NH (C-index = 0.7), see Fig. 2.

**Fig. 3:**
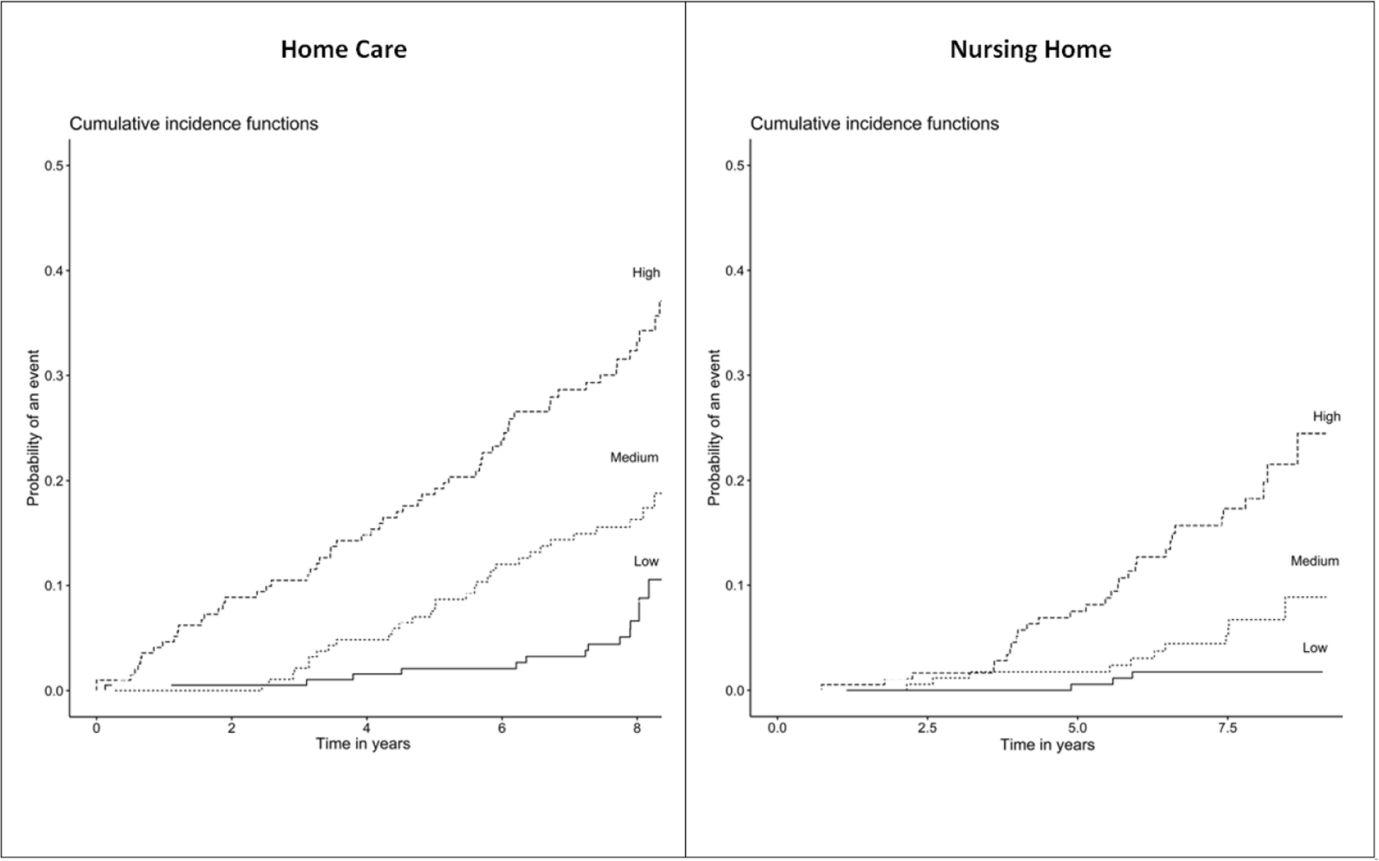
Cumulative incidence for the prediction groups of Home Care and Nursing Home events

Figure 3 depicts the cumulative incidence for each of these prognostic groups for the two events. For a HC in the lower risk group the cumulative incidence was 10.0 per 1000 patients-year; for the medium risk one it was 25.6 per 1000 patients-year; and for the higher risk one 63.1 per 1000 patients-year (*p* < 0.001). For a NH in the lower risk group the incidence was 1.9 per 1000 patients-year; the medium risk one was 4.2 per 1000 patients-year; and the higher risk one was 32.9 per 1000 patients-year (p < 0.001).

## Discussion

### Incidence of home care and nursing home entry

In this eight-year cohort study, a follow-up of more than 95% participants was obtained, with 19% entering home care programmes, and 8% geriatric nursing homes, outcomes that are generally associated with advanced frailty, especially when not addressed promptly and adequately. Health service utilization is influenced by numerous aspects, including sociodemographic, organizational, and sociocultural ones. Nevertheless, it is beyond the scope of this paper to compare HC/NH rates among countries or different health systems. We used, instead, these events as measures of health outcomes associated with the loss of functionality and independence. However, regarding HC, although difficult to compare, similar rates were observed between homebound incidence in Japan [41] (32.1 per 1000 individuals/year) and that of our study (30.6 per individual/year). With respect to NH entry, we found lower rates than those reported by the USA [42], 16.1% in 2 years; but closer to Germany, with rates of 4.7% in a three-year follow-up [43]. Again, institutionalization rates differ among countries, depending on organizational aspects, the availability of long-term beds and the responsibility for the care of disabled older individuals by different actors. Our rates of institutionalization are low, this could be explained by the fact that in southern European cultures the involvement of the family in the care of older adults is considerable, whether for cultural or economic reasons [44, 45].

### Risk factors

#### Age and sex

In our results, increasing age was the main predisposing factor associated with frailty and both an HC and NH placement. Although women had a higher incidence of NH inclusion, it was not a statistically significant predictor. The higher life expectancy of women, and the greater percentage of their living alone, could explain this trend [46].

#### Functional status

We observed that IADL deterioration was associated with both a HC and NH. The ability to perform instrumental activities of daily life autonomously is essential to live at home independently. It is, therefore, a relevant measure to take into account when predicting the path to functional decline and dependence. We found that even mild dependence in the IADL was associated with a future HC, and moderate to severe dependence with NH entry. IADL impairment has been described as a potential marker of frailty [47], implying losses in different functioning domains [48]. There is, however, controversy with respect to disability and its inclusion in the definition of frailty [49]. Nevertheless, early stages of IADL impairment could be useful in detecting individuals at risk, it is an easy measure to collect and has a long-established tradition in PHC settings. Our sample included very few subjects with dependence in basic activities of daily living as it was composed of community-dwelling, independent individuals. BADL thus had no impact on the prediction models.

#### Mobility

We measured mobility with the TUGT. It has been shown to have high sensitivity for identifying frailty [11], moreover, as it is a simple test requiring little equipment and space, the TUGT is a valuable tool in a clinical setting. Savva et al. [50] found that a cut-off point of more than 16 s was optimum to identify the frail population. Our results concur, we observed a mean score of 16.2 s for subjects entering a HC programme, and 15.5 s for a NH one. Due to the fact that the TUGT has been used as a proxy measure of frailty [50] and subsequent functional decline, it is a relevant factor in a HC prediction model.

#### Polypharmacy

Polypharmacy is a measure of medication-associated frailty, irrespective of the number of comorbidities and their severity [51]. It is associated with increased rates of falls [52] and hospitalization, disability, and mortality [53]. In our sample, polypharmacy, collected from the electronic primary healthcare records, was a prevailing factor. Some 52% of the participants had more than 4 prescribed drugs which was higher than in other studies reporting a prevalence based on health surveys of between 26 and 40% [54]. It was, however, closer to those authors employing electronic healthcare records [55] who observed over 50%. In our study, polypharmacy predicted NH entry, a fact that might be related to multimorbidity in addition to adverse drug reactions/interactions, and greater risk of falls, and negative health outcomes [56].

Particular emphasis should be placed on psychoactive drugs as 50% of those entering a NH were taking them. Moreover, inappropriate polypharmacy is a key issue to address in order to improve outcomes in older adults [57] by means of active medication reviews and deprescription processes with tools such as STOPP START criteria and other available strategies [58].

#### Social vulnerability

We observed that whilst living alone had no effect on the need for an HC it did influence NH entry (sHR = 2.8), a finding that has been already described in other countries [59–62]. Employing an exhaustive socio-family situation measure, the SFRSE scale, we found a strong association between greater social risk and a higher institutionalization rate, irrespective of functional status or comorbidities. It appears as one of the predictors in a NH entry equation highlighting the importance of social support and environment in maintaining the capacity to live in the community in one’s home. Social and caregiver networks could help circumvent institutionalization, as has been observed in various studies that only take either living arrangements or caregiver networks into account [63, 64]. The need to assess the social sphere of frail older adults is evident [65]. Indeed, as the issue of social frailty is increasingly conceptualized [66], the design of interventions to improve social support resources and promote inclusion of older adults will become essential in granting their preferences for living in the community, and thus improving quality of life.

#### Other factors

Cognitive impairment and dementia are factors classically described as being related to NH placement [61]. Whilst we observed a bivariate association between cognitive status and adverse events it was not included in the final model. This was due to the low prevalence of dementia in our free-living, community-dwelling population. As we lacked a longitudinal measure of the incidence of cognitive impairment in our sample, we could not test the association with enough statistical power. The same pattern of bivariate association was also reported for depression, nutritional risk, and urinary incontinence. The latter is additionally usually found as a strong gender-specific predictor [67], negatively affecting daily life although our final model did not include it.

#### Strengths and limitations

Few longitudinal studies can be found in the literature analysing the transition of frail older adults from the commencement of their requiring home care to later nursing home placement, both outcomes related to functional decline and loss of autonomy. Our cohort had an excellent follow-up rate, up to 95% of the sample, and was representative of the older patients attended in primary healthcare in Catalonia, around 12.6% of the total patient population. Although our external validity was limited to those who sought medical assistance at the PHC it should be noted that this was not a health survey aimed at representing all the older population in this region. Moreover, most of these individuals in Spain seek medical assistance in the public health sector.

Despite the fact that our models included the main CGA variables there might have been other factors influencing an HC/NH admission. Nevertheless, the CGA is comprised of the most important known dimensions, and they were measured using standardised, validated questionnaires and scales. Finally, although an extensive follow-up was performed, changes in baseline variables during follow-up were not analyzed as it was a prediction model based on the initial situation of the sample.

## Conclusions

Prognostic models established with comprehensive geriatric assessments can predict the commencement of the need for HC and subsequent NH entry in community-dwelling, older adults. Our findings underline the necessity to measure functional capacity, mobility, inappropriate prescriptions, and social aspects of older adults in primary care settings where they can be offered holistic, longitudinal assessments, and tailored interventions.

Such models could also be useful for the risk classification of frail older adults and in the planning of health care policies.

### Recommendations

- Due to the relevance of mobility and instrumental activities of daily living in the prediction of adverse outcomes, community interventions based on physical and functional exercises should be prioritised to improve/maintain independence and quality of life in older adults [68, 69].
- Tackling polypharmacy and inappropriate prescriptions through deprescription processes at the primary care level should also be prioritised [69, 70].
- Interventions to improve social resources and promote social support networks and inclusion in the community would improve the quality of life of older adults. Moreover, they would enhance the efficiency of the health system and, given the high cost of residential centres, ease the financial burden for both for families and society [70].

## Data Availability

The dataset supporting the conclusions of this article is available in the Open Science Framework repository, in [https://osf.io/sqty8/].

## Abbreviations

BADL: Basic Activities of the Daily Living
BMI: Body mass index
CIF: Cumulative incidence function
CGA: Comprehensive Geriatric Assessment
GDS: Geriatric Depression Scale
HC: Home care
HHIE-S: Handicap Hearing Impairment in the Elderly Screening Version
IADL: Instrumental Activities of the Daily Living
ICIQ-SF: International Consultation on Incontinence Questionnaire Short Form
IQ: Interquartile
MCE: Mini Cognitive Examination
MNA-SF: Mini Nutritional Assessment Short Form
NH: Nursing home
PHC: Primary Health Care
SFRSE: Socio-Family Rating Scale of the Elderly
sHR: Subdistribution hazard
TUGT: Timed-up-and-go test
